# Influence of Silica Fume Addition in the Long-Term Performance of Sustainable Cement Grouts for Micropiles Exposed to a Sulphate Aggressive Medium

**DOI:** 10.3390/ma10080890

**Published:** 2017-08-02

**Authors:** José Marcos Ortega, María Dolores Esteban, Raúl Rubén Rodríguez, José Luis Pastor, Francisco José Ibanco, Isidro Sánchez, Miguel Ángel Climent

**Affiliations:** 1 Departamento de Ingeniería Civil, Universidad de Alicante, Ap. Correos 99, 03080 Alacant/Alicante, Spain; joseluis.pastor@ua.es (J.L.P.); fjis@alu.ua.es (F.J.I.); isidro.sanchez@ua.es (I.S.); ma.climent@ua.es (M.Á.C.); 2 Departamento de Ingeniería Civil, Urbanismo y Aeroespacial, Escuela de Arquitectura, Ingeniería y Diseño, Universidad Europea, c/Tajo s/n, Villaviciosa de Odón, 28670 Madrid, Spain; mariadolores.esteban@universidadeuropea.es (M.D.E.); raulruben.rodriguez@universidadeuropea.es (R.R.R.)

**Keywords:** micropiles, sustainability, silica fume, special geotechnical works, impedance spectroscopy, microstructure, compressive strength, sulphate attack, cement grouts

## Abstract

At present, sustainability is of major importance in the cement industry, and the use of additions such as silica fume as clinker replacement contributes towards that goal. Special foundations, and particularly micropiles, are one of the most suitable areas for the use of sustainable cements. The aim of this research is to analyse the effects in the very long-term (for 600 days) produced by sulphate attack in the microstructure of grouts for micropiles in which OPC (ordinary Portland cement) has been replaced by 5% and 10% silica fume. This line of study is building on a previous work, where these effects were studied in slag and fly ash grouts. Grouts made using a commercial sulphate-resisting Portland cement were also studied. The non-destructive impedance spectroscopy technique, mercury intrusion porosimetry, and Wenner resistivity testing were used. Mass variation and the compressive strength have also been analysed. Apparently, impedance spectroscopy is the most suitable technique for studying sulphate attack development. According to the results obtained, grouts for micropiles with a content of silica fume up to 10% and exposed to an aggressive sulphate medium, have a similar or even better behaviour in the very long-term, compared to grouts prepared using sulphate-resisting Portland cement.

## 1. Introduction

Recently, the use of additions as clinker replacement in cement manufacturing has become very common [1,2,3,4,5]. Among the benefits provided by the additions, it is important to emphasise their contribution to cement industry sustainability, because they reduce the CO_2_ emissions generated during the cement production. Moreover, the majority of these additions are residues from other industrial processes, and their reuse is also a benefit. On the other hand, some of them are active additions, which means that they can react with water or with Portlandite that has been produced as a product of hydration reactions of clinker. This reaction forms new hydrated products that boost the cement-based materials’ properties [6,7,8,9,10]. The most popular active additions are fly ash, silica fume, and ground granulated blast furnace slag.

In relation to silica fume, research indicates that this addition improves the microstructure and properties of cement-based materials [11,12,13,14], such as their permeability, and recommend its use for several applications. However, at least in Spain, there are several fields of civil engineering where silica fume is not used. One of these fields is special geotechnical works, which have experienced great development. Among them, micropiles are very popular at present. Micropiles are frequently used to transfer loads from structures to deep strata when shallow soil is too soft to support those loads. They consist of small-diameter piles with diameters under 300 mm that are drilled and grouted with cement grout or mortar, and then reinforced with steel tubing and sometimes with rebars [15,16,17].

The micropiles’ standards [15,16,17] do not specify the cement type to use for preparing the grouts, provided that they reach a certain compressive strength. Then, the micropiles’ grouts could constitute a potential area in order to extend the use of sustainable cement mixes that incorporate silica fume. Nevertheless, especially in Spain, the cement grouts for micropiles are usually prepared with ordinary Portland cement (OPC), without any addition.

Regarding the durability of micropiles, it is important to insist that the reinforcement elements of this special geotechnical work are embedded in cement grouts instead of concrete, as in most of civil engineering and building structures. Then, these elements could differently be affected by the attack of aggressive substances, especially if sustainable cement with an active addition, such as silica fume, is used.

A very common aggressive substance present in soils and groundwater is the sulphate ion; therefore, the micropiles can be exposed to their attack. The attack produced by sulphate ions is complex [18]. In the first place, Portlandite progressively is dissolved and a CSH phases decomposition is also produced [18,19]. The following step is the formation of expansive gypsum and ettringite crystals. These crystals occupy the pores, and when they are filled enough, they start to cause volumetric strains in the material [20], which produce microcracking and a loss of pore refinement. Finally, the gradual development of the sulphate attack entails a reduction of mechanical properties and a loss of durability [21]. The chemical reactions produced by the sulphate attack are shown in Equations (1) and (2).

Ca(OH)_2_ + Na_2_SO_4_ + 2H_2_O → CaSO_4_·2H_2_O + 2NaOH (1)

3CaO·Al_2_O_3_ + 3CaSO_4_·2H_2_O + 26H_2_O → 3CaO·Al_2_O_3_·3CaSO_4_·32H_2_O (2)

On that subject, the positive effect of silica fume on sulphate attack resistance is relatively well known [22,23]. It has been suggested that this good performance could be due to the pozzolanic reaction of silica fume consuming the Portlandite (calcium hydroxide) present in the cementitious materials before it can react with the sulphate ions in the solution [22,23,24]. As a consequence of the lower availability of calcium hydroxide, the reaction shown in Equation (1) does not take place to the same extent. Therefore, much less expansive ettringite is formed (see Equation (2)). In addition to this, regarding the specific attack by sodium sulphate, several authors [24,25] have reported the possible beneficial role that Na_2_SO_4_ could play as an activator of pozzolanic reactions of silica fume, which would more quickly reduce the Portlandite existing in the cementitious system.

With respect to sustainable grouts for micropiles exposed to sulphate attack, recent research [26] has observed a good long-term performance of grouts prepared with cements that incorporate fly ash and ground granulated blast furnace slag, in relation to their pore structure and mechanical properties. Furthermore, in that work [26], the evolution of the grouts’ microstructure in contact with the aggressive sulphate media was successfully followed with the novel non-destructive impedance spectroscopy technique for an exposure period of up to 600 days. This research [26] was the first experience in which this technique was used for studying the effects of long-term sulphate attack on the microstructure when sustainable cements are used.

On the other hand, it is interesting to note another recent work in relation to the study of the microstructure of cement-based materials that incorporate silica fume exposed to sulphate attack using the novel non-destructive impedance spectroscopy technique [24]. In that research, mortars with 5% and 10% of silica fume were kept in different sulphate media for 90 days, and their microstructure was also adequately characterised with impedance spectroscopy.

The present research continues both abovementioned works [24,26], but now the main aim is to analyse the effects in the very long-term (until 600 days) of sulphate attack in the microstructure of grouts for micropiles, in which ordinary Portland cement has been partially replaced by silica fume. As a reference, grouts made using a commercial sulphate-resisting Portland cement were also studied. The pore network evolution of the grouts has been followed with the non-destructive impedance spectroscopy technique, and its results were checked with those obtained using the classical destructive mercury intrusion porosimetry technique and the well-known non-destructive Wenner resistivity test. The changes of compressive strength and the mass variation of the grouts through the 600-day period have also been studied, due to their significance in checking the performance of cement-based materials exposed to sulphate media [18,23,27,28] and their relevance in relation to the requirements of micropiles standards [15,16,17].

## 2. Materials and Methods

### 2.1. Sample Preparation

Three types of cement grouts were studied in this research. The first one was prepared using a commercial sulphate-resisting Portland cement, CEM I 42.5 R/SR [29] (CEM I SR hereafter). Furthermore, two sustainable blended cements that incorporate silica fume were used. Both blended cements were made with an ordinary Portland cement, CEM I 42.5 R [29], which was replaced by 5% and 10% of silica fume, from now on referred to as SF5 and SF10, respectively. The water to cement ratio was 0.5 for all the grouts, which is in line with the requirements of micropiles standards [15,16,17]. The silica fume used was undensified, with a cumulative volume of particles under 1 µm of 90.78%, and an average particle diameter of 0.67 µm.

Several kinds of samples were made. On the one hand, two types of cylindrical specimens were prepared and cast in moulds of 10 cm diameter and 15 cm height, and 7.5 cm diameter and 30 cm height, respectively. On the other hand, prismatic specimens with dimensions 4 cm × 4 cm × 16 cm were also made [30]. All the samples were cured for seven days in a temperature and humidity-controlled chamber at 20 °C and 95% RH. When this curing procedure had finished, they were demoulded and the 4 cm × 4 cm × 16 cm specimens were cut in three samples of dimensions 4 cm × 4 cm × 5.3 cm. Moreover, the 15 cm-height cylindrical samples were also cut to obtain discs of approximately 2 cm thickness. After that, all the samples were exposed to the aggressive medium.

### 2.2. Exposure Medium

Once the curing period finished, the specimens were exposed to an aggressive sulphate medium. This medium was 15% reagent grade anhydrous sodium sulphate (Na_2_SO_4_) aqueous solution by weight. In order to monitor the changes in the microstructure and the mechanical strength of the grouts in the long-term from the sulphate attack, the exposure period was extended up to 600 days. The sodium sulphate solution was replaced every 60 days during the studied period. The volume of sulphate solution was approximately four times the volume of the samples, as recommended by the ASTM C 1012-04 standard [31].

### 2.3. Impedance Spectroscopy

Among the advantages of the non-destructive impedance spectroscopy technique compared to other classical techniques, it is important to note the ability to obtain global information regarding the microstructure of the samples, as well as register the modifications of the pore network of the same sample during the studied period. This technique has recently been used for monitoring the evolution of the microstructure of cementitious materials, although most of them were prepared with ordinary Portland cement, without any addition [4,32,33,34]. In relation to the analysis of the long-term effects of sulphate attack in the microstructure of cement-based materials that incorporate active additions using impedance spectroscopy, the only experience is a recent authors’ work [26] in which OPC, fly ash, and slag cement grouts were studied for up to 600 days of exposure. On the other hand, there is another recent authors’ work regarding the study of silica fume cementitious materials with impedance spectroscopy [24], where the microstructure evolution of mortars with 5% and 10% of silica fume was studied up to 90 days of exposure to different types of sulphate attack. This research is the first experiment to use impedance spectroscopy to study cement samples with silica fume exposed to sulphate attack in the very long-term.

**Figure 1:**
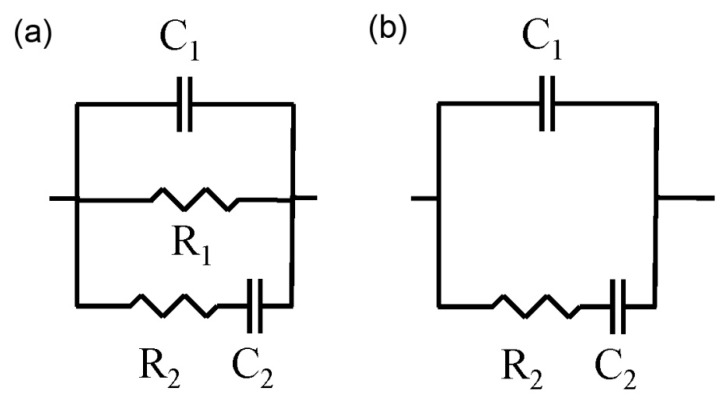
(**a**) Equivalent circuit used for the fitting of the impedance spectra obtained using the contacting method; (**b**) Equivalent circuit used for the fitting of the impedance spectra obtained using the non-contacting method.

The impedance analyser used for performing the measurements on cement grouts was an Agilent 4294A (Agilent Technologies, Kobe, Japan), which allows capacitance measurements in the range from 10^−14^ F to 0.1 F, and a maximum resolution of 10^−15^ F. The measurements were taken over a frequency range of 100 Hz to 100 MHz. For all measurements, the electrodes used were circular (Ø = 8 cm), made of flexible graphite, and attached to a copper piece with the same diameter. Both contacting and non-contacting methods were used [4,32,35]. The measured data were fitted to the equivalent circuits proposed by Cabeza et al. [32] (see Figure 1), which include two time constants. Those circuits are constituted by several resistances and capacitances, which are related to different elements of the microstructure of cement-based materials. On the one hand, the resistance R_1_ is associated only with the percolating pores of the sample, while the resistance R_2_ provides information about all its pores. On the other hand, the capacitance C_1_ is related to the solid fraction of the sample, and the capacitance C_2_ is associated to the pore surface in contact with the electrolyte, which fills the pore network of the material.

In one of the previous authors’ recent work [24], the validity of these equivalent circuits for silica fume cement-based materials was already checked using the Kramers–Kronig (K–K) relations [36]. The impedance parameters R_2_, C_1_ and C_2_ are present in both contacting and non-contacting methods (see Figure 1). Here, only the values of these parameters obtained with the non-contacting method have been studied, due to its higher degree of accuracy.

Five different slices of approximately 2 cm thickness were tested for each cement type. The evolution of impedance parameters with time has been reported over a 600-day exposure period.

### 2.4. Electrical Resistivity

The electrical resistivity provides information related to microstructure pore connectivity in cementitious materials [37,38]. The resistivity of the grouts was obtained using the non-destructive Wenner four-point test, according to the Spanish standard UNE 83988-2 [39]. This parameter was measured using a Proceq analyser on 30 cm-height and 7.5 cm-diameter cylinders along the 600 exposure days to sulphate medium.

### 2.5. Mercury Intrusion Porosimetry

In order to check the results of the non-destructive techniques, the microstructure of the mortars was also studied using mercury intrusion porosimetry. This test was performed with a Micromeritics Autopore IV 9500 porosimeter (Norcross, GA, USA). The samples were dried in an oven for 48 h at 50 °C prior to the test. They were obtained from discs of 2 cm-height. For each age, two measurements were performed on each grout type. Total porosity, pore size distribution and the percentage of mercury (Hg) retained at the end of the experiment were studied. The testing ages were 28, 60, 90, 120, 180, 365, and 600 days.

### 2.6. Mass Variation

The monitoring of samples’ mass changes is frequently used for studying the behaviour of cementitious materials exposed to sulphate attack [40,41]. Here, the mass variation has been tracked up to 600 days of exposure to sodium sulphate solution in 4 cm × 4 cm × 5.3 cm specimens, which were also used to study the compressive strength. Specifically, the percentage of mass variation with respect to the initial mass of the samples, which was measured before exposing them to the sodium sulphate solution after the seven-day curing period, was analysed.

### 2.7. Compressive Strength

The compressive strength is a commonly used parameter for following the attack produced by sulphates in cement-based materials [18,42,43]. Furthermore, the micropiles standards [15,16,17] require a certain compressive strength of cement grouts, so it is interesting to analyse this parameter in regards to the effects of sulphate attack. In this research, the compressive strength was determined in prismatic samples of dimensions 4 cm × 4 cm × 5.3 cm, according to the Spanish standard UNE-EN 196-1 [30]. Three specimens were tested for each cement type at the ages of 28, 60, 90, 120, 180, 365, and 600 days.

## 3. Results

### 3.1. Impedance Spectroscopy

**Figure 2:**
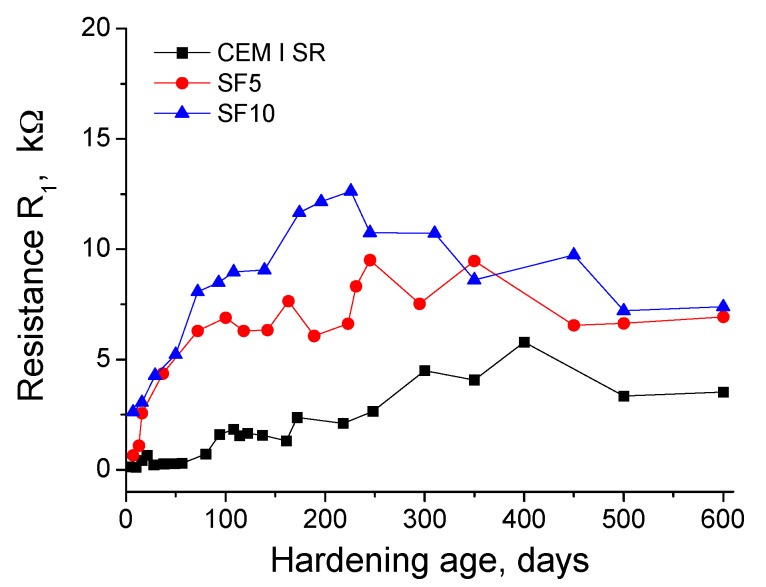
Results of impedance spectroscopy resistance R_1_ for grouts prepared using sulphate-resisting Portland cement (CEM I SR), and for those with a content of 5% (SF5) and 10% (SF10) of silica fume.

The results of resistance R_1_ are depicted in Figure 2. In general, this parameter rose in the short-term for the studied grouts. The highest R_1_ values were observed for silica fume grouts during the entire exposure period, which reached their maximums between 200–250 hardening days, depending on the content of addition. Moreover, the R_1_ increasing rate was also greater for those grouts compared to CEM I SR ones. Since the abovementioned ages, the resistance R_1_ of silica fume grouts showed a slight decrease until 600 days, which was more noticeable for those with 10% silica fume (SF10). For CEM I SR, the R_1_ increase was slower, with a maximum at approximately 400 days, and scarcely falling for up to 600 days.

**Figure 3:**
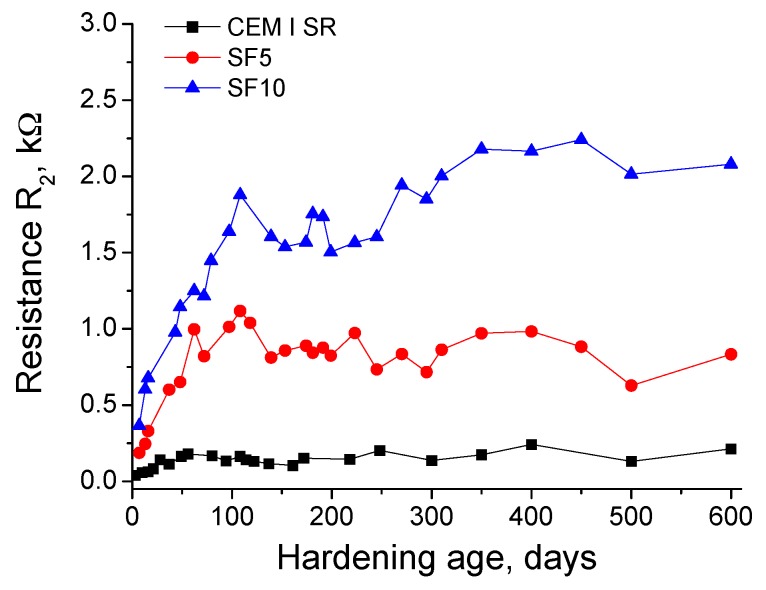
Results of resistance R_2_ for CEM I SR, SF5 and SF10 grouts.

In relation to resistance R_2_, results can be observed in Figure 3. Overall, there are similarities with those obtained for resistance R_1_, although the difference between grout types is more marked. The grouts with 10% silica fume (SF10) had the greatest R_2_ values throughout the studied time. For that type of grout, the fastest rise of this parameter was observed until 150 days; which then slowed down between exposure days 150–600. The R_2_ evolution for 5% silica fume (SF5) grouts was very similar to that described for SF10 grouts, although the R_2_ values of the SF5 group were lower. The smallest R_2_ corresponded to CEM I SR grouts, in which this parameter hardly changed with time.

**Figure 4:**
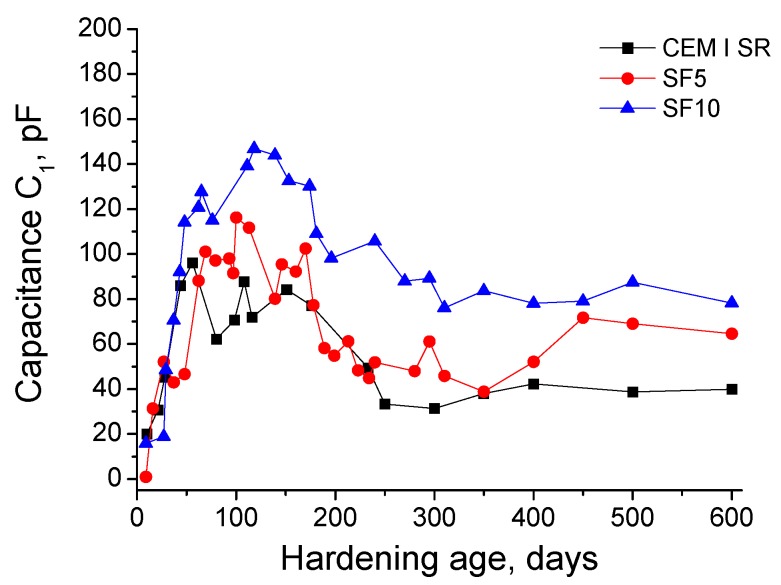
Impedance capacitance C_1_ results for CEM I SR, SF5 and SF10 grouts.

The capacitance C_1_ results are represented in Figure 4. In the short-term, this capacitance increased for all the grouts independently of the cement type used. However, the age at which they reached a maximum C_1_ value was different, depending on the kind of grout. Particularly, the C_1_ maximum was observed at around 100 exposure days for silica fume grouts, and at approximately 50 days for CEM I SR grouts. Furthermore, until that age, the capacitance C_1_ values were very similar for the three analysed grouts. Since then, the greatest capacitance C_1_ values were observed for SF10 grouts, although this parameter in general showed a similar magnitude for all the grouts studied. After reaching their respective C_1_ maximums for each grout type, this parameter gradually fell over the remainder of the 600-day period.

**Figure 5:**
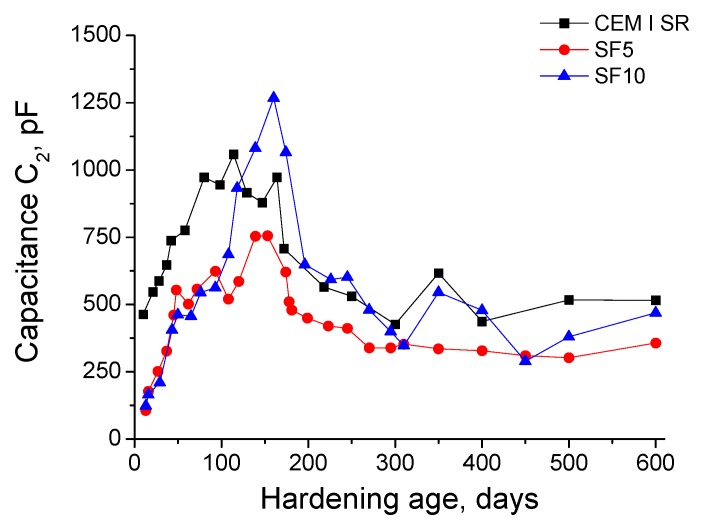
Results of capacitance C_2_ for CEM I SR, SF5 and SF10 grouts.

The results for the impedance spectroscopy capacitance C_2_ are depicted in Figure 5. As with capacitance C_1_, the evolution of C_2_ was very similar regardless of the grout kind, showing a similar magnitude in general across all of the grout types. This parameter rose for approximately 150 days, and decreased afterwards. At early ages, the highest capacitance C_2_ corresponded to CEM I SR grouts, and this parameter was lower and very similar for both types of silica fume grouts. However, after 100 days, the C_2_ growth of SF5 grouts tended to stabilise, while the C_2_ values for SF10 continued increasing, and even overtook those for CEM I SR grouts. In general, the capacitance C_2_ did not show great differences between the three types of grouts between days 200 and 600, although it was slightly higher for CEM I SR and SF10 grouts than for SF5 grouts.

### 3.2. Electrical Resistivity

**Figure 6:**
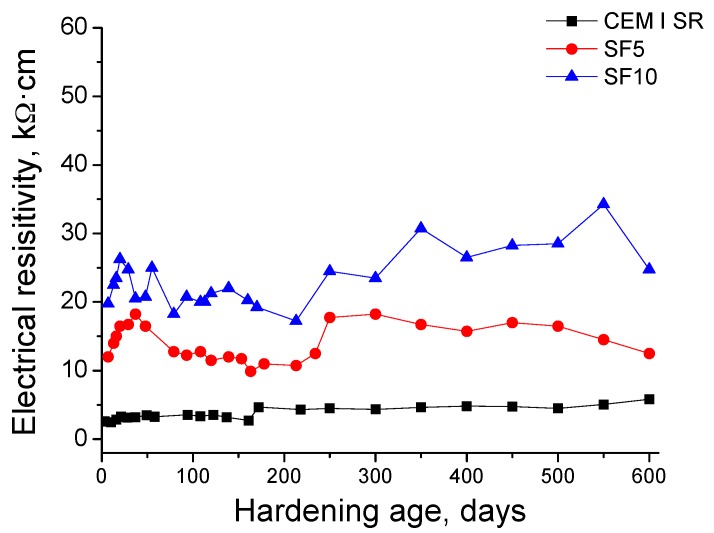
Evolution of electrical resistivity for CEM I SR, SF5 and SF10 grouts.

The results of electrical resistivity obtained for the three types of grouts studied are depicted in Figure 6. The highest values of this parameter were observed for SF10 grouts, followed by SF5. The electrical resistivity showed an increase in the short-term (until 50 days, approximately) for both silica fume grouts. From the next 50 days it decreased, after which the resistivity kept constant up to 250 days, when it rose again. Finally, it hardly changed after 250 days, until the end of studied exposure period. On the other hand, the grouts made using CEM I SR showed the lowest electrical resistivity values, which also barely varied with hardening age.

### 3.3. Mercury Intrusion Porosimetry

**Figure 7:**
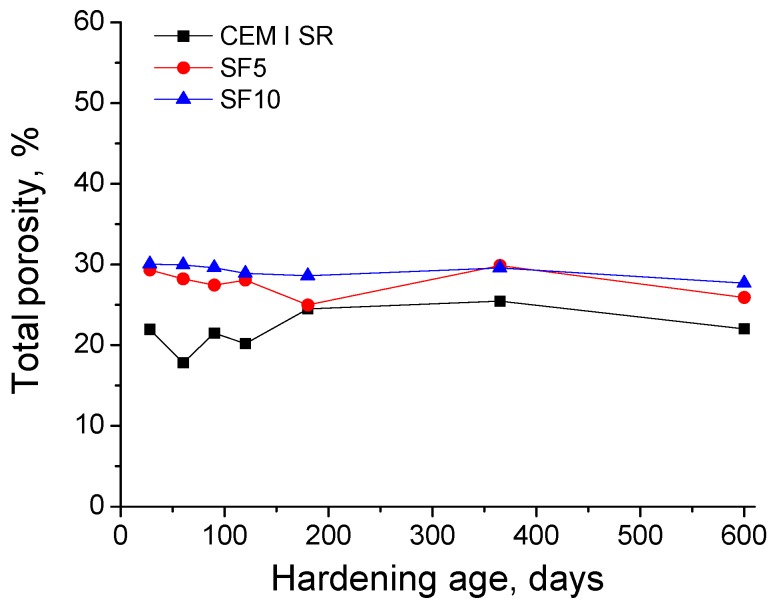
Total porosity results for CEM I SR, SF5 and SF10 grouts.

The evolution of total porosity for the three kinds of studied grouts is represented in Figure 7. This parameter showed a similar magnitude for all the grouts independently of the cement type used. The total porosity kept practically constant during the exposure period for both silica fume grouts, and its values were almost identical at the majority of testing ages. For CEM I SR grouts, this parameter was slightly lower compared to silica fume grouts, and it also hardly changed with hardening age.

**Figure 8:**
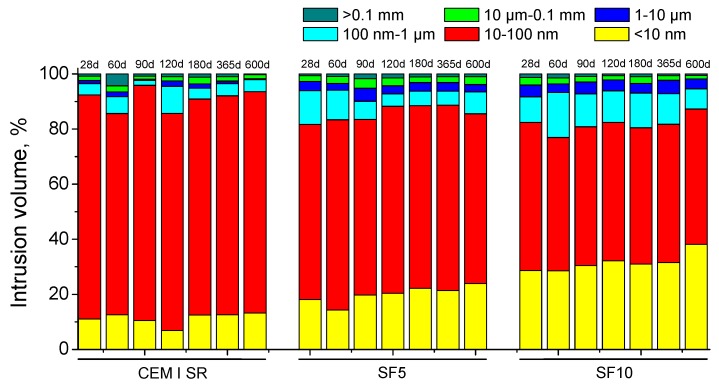
Pore size distributions (in percentage) for CEM I SR, SF5 and SF10 grouts.

The pore size distributions obtained for CEM I SR, SF5 and SF10 exposed to the sulphate aggressive medium are shown in Figure 8. In general, the main pore size range for the three studied grouts is between 10 and 100 nm. It is important to point out that the percentage of pores with a size less than 10 nm was higher for silica fume grouts (especially the SF10 type) than for CEM I SR grouts. Nevertheless, the percentage of pores with diameters smaller than 100 nm was greater for CEM I SR grouts than for silica fume grouts. Therefore, the pore network of silica fume grouts was more refined, although in global terms the degree of microstructure refinement showed by CEM I SR grouts was not considerably lower than silica fume grouts. In relation to the evolution of pore size distributions, a progressive pore refinement was observed over the exposure time for all the studied grouts overall, as suggested by the increase in the relative volume of smaller-diameter pores.

**Figure 9:**
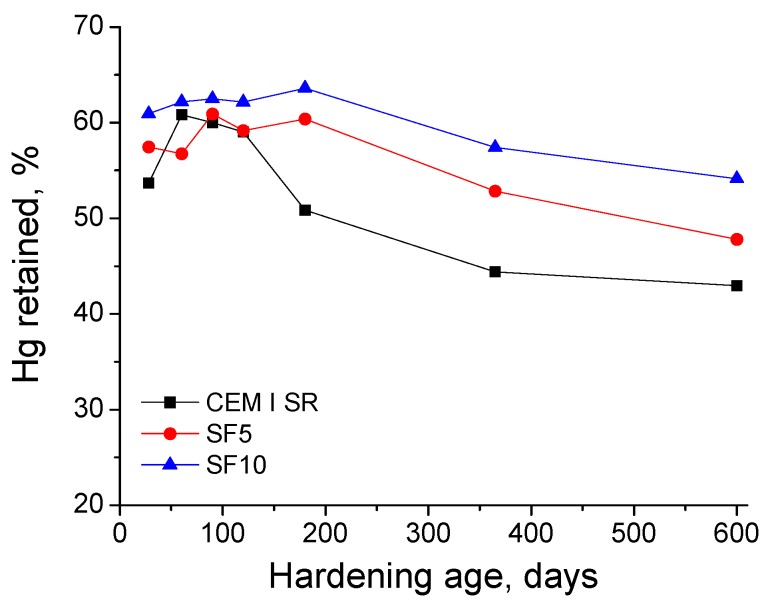
Percentage of Hg retained at the end of experiment for studied grouts.

As for the percentage of Hg retained in the samples at the end of the experiment, see Figure 9. The greatest percentage corresponded to SF10 grouts at all exposure ages. For that type of grout, the Hg retained showed a slight rise from 28 to 200 days, after which it fell until day 600. The evolution of this parameter for SF5 grouts was very similar to that described for SF10 ones, although the Hg-retained values of SF5 grouts were lower. On the other hand, for CEM I SR grouts, this parameter increased between 28 and 90 days, and decreased thereafter. Until day 120, the values observed for that kind of grout were similar to those noted for the SF5 type. However, at day 180, 365 and 600, the Hg retained by CEM I SR grouts was the lowest of all the analysed samples.

### 3.4. Mass Variation

**Figure 10:**
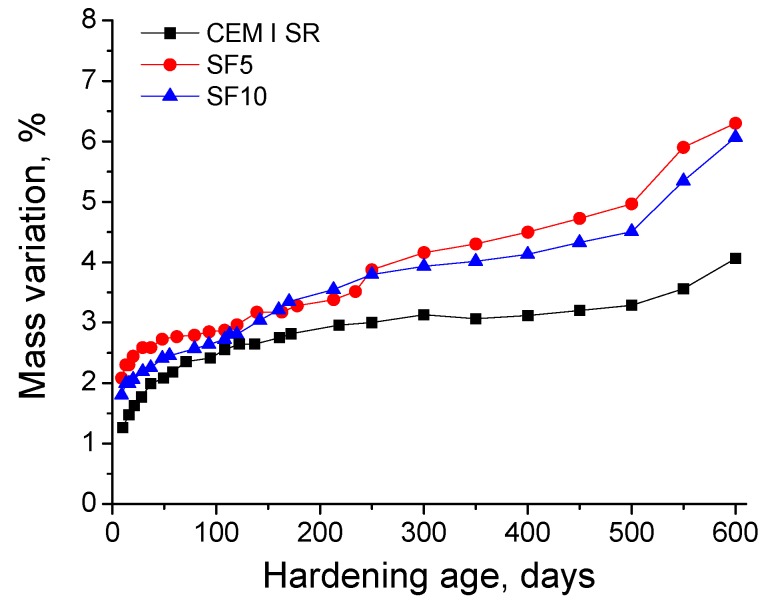
Mass variation (in percentage) observed for CEM I SR, SF5 and SF10 grouts in contact with the aggressive medium.

Regarding the mass variation of the grouts produced by contact with the aggressive sulphate medium, the evolution of this parameter over time is depicted in Figure 10. As can be observed, this parameter showed a progressive increase for all the grouts during the studied time period. The mass variation percentages were higher for silica fume grouts than for CEM I SR ones.

### 3.5. Compressive Strength

**Figure 11:**
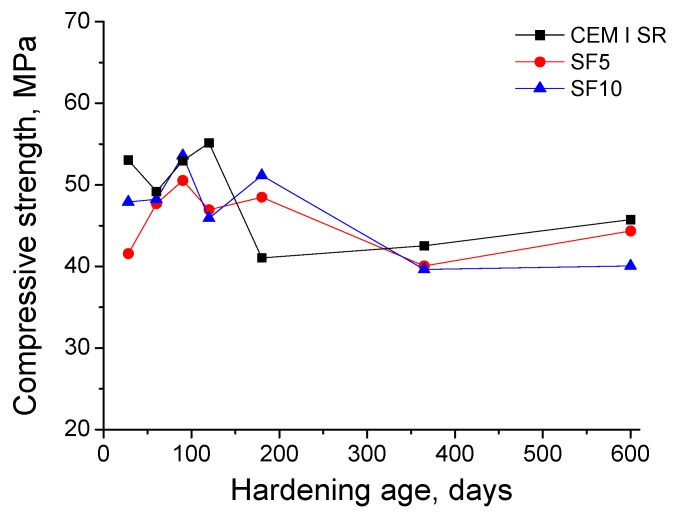
Results of compressive strength obtained for CEM I SR, SF5 and SF10 grouts.

The compressive strength results of the grouts can be observed in Figure 11. In the first place, for CEM I SR grouts, this parameter showed a slight reduction between days 28 and 60, increased between days 61 and 120, fell at day 180, and experienced a slight rise between days 181 and 600. The compressive strength of SF5 grouts rose for the first 90 days, remained constant between days 91 and 180, and decreased during the rest of studied period. In general terms, the strength for SF10 grouts grew for the first 180 days, except for a drop at day 120, and fell between days 180 and 600. Overall, there were small differences between the compressive strength values showed by the three types of grouts. At 28 days, the highest compressive strength corresponded to the CEM I SR grouts, and the lowest was observed for the SF5 grouts. This parameter was similar for all the grouts at day 60 and 90. However, it was greater for CEM I SR grouts at 120 days, and for silica fume grouts at 180 days. Finally, the compressive strengths in the very long-term (365 and 600 days) hardly differed between the analysed grouts.

## 4. Discussion

In the first instance, with respect to impedance spectroscopy results, both resistances R_1_ and R_2_ are related to the electrolyte that fills the pores of the sample [32]. Then, the evolution of those parameters give information about the microstructural changes of the grouts, produced by either the development of hydration and pozzolanic reactions, or the progress of the sulphate attack.

The resistance R_1_ is associated only with the percolating pores of the sample [32]. The rise of this parameter in the short-term and in the middle-term (see Figure 2), which has been observed for all grout types, could be due to the progressive pore network refinement, produced by the development of clinker hydration and silica fume pozzolanic reactions. Since the sulphate attack is beginning during those relatively early stages, as a consequence, practically no deleterious effects of the attack were still observed in the pore network. The higher R_1_ obtained for silica fume grouts compared to CEM I SR grouts would indicate that silica fume grouts have a more refined microstructure [4,24]. Moreover, the fact that the resistance R_1_ was slightly higher for SF10 (10% silica fume) grouts than for SF5 grouts (5% silica fume) would suggest that the pore network becomes more refined as the percentage of silica fume increases, which is in accordance with previous studies [24,44].

On the other hand, the faster R_1_ increasing rate observed for silica fume grouts could be related to the possible role of Na_2_SO_4_ as an activator for the pozzolanic reactions [24,25]. As a consequence, these reactions would develop faster in the silica fume grouts, and their products would increase the volume of the finer pores of the material. This has been suggested by other authors [44], and would also agree with resistance R_1_ results. The fall in the long-term of this parameter for all the grouts would indicate a loss of solid fraction and pore network refinement. This loss was possibly produced by the formation of expansive products during the sulphate attack [18,19,21,45], which would crack the current microstructure.

The impedance spectroscopy resistance R_2_ value provides information about all the pores of the sample [32]. Generally, the results of this parameter (see Figure 3) are in agreement with resistance R_1_ results. Again, the highest R_2_ values corresponded to silica fume grouts, which would suggest that their microstructure was more refined than that of the CEM I SR grouts. Furthermore, as also happened with resistance R_1_, when the silica fume percentage was higher, R_2_ values were higher, which would corroborate findings regarding the influence of this addition on the pore size distribution of cement-based materials [24,44]. Another coincidence between resistances R_1_ and R_2_ was their quick rise at early ages. The R_2_ results also reveal the previously mentioned possible effect of Na_2_SO_4_ as an activator for the pozzolanic reactions [24,25].

However, several differences were noted in the resistance R_2_ results compared to those obtained for R_1_. On the one hand, the gap between cement types was higher in relation to R_2_ values than to those of R_1_. On the other hand, there was hardly a drop in the very long-term of resistance R_2_ for all the grouts, while a slightly more noticeable fall was observed for resistance R_1_. Similar differences between resistances R_1_ and R_2_ were obtained in a previous recent authors’ work [26], in which the evolution in the long-term of the microstructure of slag and fly ash cement grouts exposed to sulphate attack was studied with impedance spectroscopy. In that research [26], the different behaviour of both resistances was related to the fact that the resistance R_1_ is associated with the percolating pores of the sample [32], whereas the resistance R_2_ gives information about both the occluded and percolating pores of the sample [32]. The sulphate attack would develop sooner in the percolating pores because they are directly accessible for the aggressive sulphate solution. Their effects would be first detected through resistance R_1_, which would corroborate with its decrease at later stages. Moreover, the initial steps of sulphate attack could be done simultaneously alongside the hydration and pozzolanic reactions, so that no effects of this attack in resistance R_1_ were observed in the short-term.

On the contrary, the occluded pores would not directly receive the sulphate attack, and this would result in no damages from the attack. Therefore, in these pores the main process that is developed would be the formation of new solid phases as products of clinker hydration and silica fume pozzolanic reactions [26]. This would imply a rise of the resistance R_2_ together with the analogous process produced in percolating pores, which also contributes to increasing R_2_ values. Both would then counteract the probable falling effect in R_2_ produced by the possible damages to the percolating pores caused by the sulphate attack. This could justify the scarce differences observed in the results of impedance resistances R_2_ and R_1_ in the context of this research.

Regarding the impedance capacitance C_1_ results (see Figure 4), this parameter is associated with the solid fraction of the sample [32]. The increase in the short-term of capacitance C_1_ for all the studied grouts is in keeping with already discussed resistances R_1_ and R_2_ results, and could be due to the formation of new solid fraction as a result of the clinker hydration and silica fume pozzolanic reactions [4,24,44,46,47]. The gradual fall of capacitance C_1_ observed at later stages would denote a loss of solid fraction, which would agree with resistance R_1_ results in the long-term. This could be related to the effects of the sulphate attack, as has been already explained. Overall, for the different types of studied grouts, the capacitance C_1_ showed a similar magnitude, which would indicate that all of them would have a similar solid fraction, regardless of their pore size distribution. Then, only small porosity differences among them could be expected. This prediction was confirmed by the results obtained in the total porosity measurements, see Figure 7.

The capacitance C_2_ results in Figure 5 showed similarities with those obtained for the rest of the impedance spectroscopy parameters, especially for the resistance R_1_ and capacitance C_1_, as previously discussed. The capacitance C_2_ is associated with the pore surface in contact with the electrolyte, which fills the pore network of the material [48]. The rising tendency of this parameter in the short-term for all the analysed grouts would indicate that the pore surface increased, probably because of the formation of CSH gel layers [48] as products of clinker hydration and silica fume pozzolanic reactions. The reduction of this parameter in the long-term is also in accordance with resistance R_1_ and capacitance C_1_ results. In the previously cited authors’ work [26], which studied slag and fly ash cement grouts exposed to sulphate attack, a reduction of capacitance C_2_ was also observed. In that research [26], on the one hand, this result was related to the formation of expansive ettringite and other sulphate attack products, which would progressively break the rough structures deposited on the pore surface (formed as products of the hydration and pozzolanic reactions). On the other hand, it was also pointed out that at relative high exposure times, a progressive silting of the pores could have been produced due to the formation of sulphate attack products [26]. Both processes would entail a loss of specific pore surface, and therefore a drop of the capacitance C_2_. However, it seems that in this study of CEM I SR and silica fume grouts, the main process would be the first breaking of rough structures on pore surfaces. The second process (pores silting) would imply an increase of capacitance C_1_, which was not observed in the long-term. Despite that, the lower reduction at later stages of capacitance C_1_ compared to C_2_ would suggest that the process of closing the microstructure by pores silting was also developed, but to a lesser extent than the breaking of previously formed rough structures on pore surfaces.

The highest electrical resistivity values were obtained for silica fume grouts, which is coincident with impedance spectroscopy results, as seen in Figure 6. However, the evolution of electrical resistivity for all the studied grouts was not as clear as observed for impedance spectroscopy parameters. This could be due to the difference between both techniques. Impedance spectroscopy is a global technique that permits measurements through the samples [35,49]. As a consequence, the results of electrical resistivity would mainly reveal the processes produced in the surface of the samples, which would not be representative of what was happening in their global pore network. This drawback was already reported in previous works [26,35] in which the microstructure of slag and fly ash cement grouts immersed in a sodium sulphate solution was characterised using impedance spectroscopy and Wenner four-point electrical resistivity, among other techniques. In those studies [26,35], it was concluded that the Wenner four-point test was not the most suitable technique for studying the long-term effects of sulphate attack in the pore network of slag and fly ash cement grouts; the impedance spectroscopy was more appropiate to obtain reliable information regarding that attack. Finally, in view of the resistivity results of the current research, this could be generalised for grouts made using a sulphate-resisting Portland cement and for grouts that incorporate silica fume.

In relation to mercury intrusion porosimetry, the total porosity hardly differed among the studied grouts, as seen in Figure 7. This similarity would denote that the solid fraction was similar for all of them, which is also in agreement with the similar magnitude shown by impedance capacitance C_1_. The pore size distributions of the grouts in Figure 8 showed a progressive pore network refinement with all of them hardening with age. These results coincide with those obtained in the short-term for impedance spectroscopy parameters. Those results reveal the solid formation to be a consequence of the development of clinker hydration and silica fume pozzolanic reactions [24,44], which would entail an increase of the relative volume of smaller size pores. Moreover, the pore network was more refined for silica fume grouts, which is also in keeping with the highest values of impedance spectroscopy parameters observed for those grouts, compared to the CEM I SR grouts.

The Hg retained in the specimen after the mercury intrusion porosimetry test gives information related to the tortuosity of the microstructure [32]. Generally, this parameter increased in early stages and fell in the long-term for all the studied grouts, as seen in Figure 9. This would mean that in the first place, a rise of the pore network tortuosity of the samples was produced. This rise was also in line with the progressive microstructure refinement due to the development of hydration and pozzolanic reactions, as already explained by the impedance spectroscopy and pore size distributions results. The progressive drop of Hg retained at later stages would indicate the effects of sulphate attack, which broke the rough structures formed on the pore surfaces, thus reducing the tortuosity of the pore structure, as has been discussed near the impedance capacitance C_2_ results.

Despite showing similarities with the results obtained by the other techniques used in this research, especially in the short-term, mercury intrusion porosimetry did not detect any deleterious effects of sulphate attack at later stages. Some examples of the lack of coincidence include the lack of drop of porosity, and the lack of loss of pore refinement for all the grouts in the long-term. The only exception was the slight reduction of Hg retained, which was expected in light of impedance spectroscopy results. This fact would corroborate the results observed in the previous works [24,26,35] to this research, in which mercury intrusion porosimetry results were also not as clear as those observed using impedance spectroscopy. This could be due to the limitations of mercury intrusion porosimetry [50,51], such as the inability to register pores higher than 900 µm, as well as the overestimation of smaller pores existing in the tested specimens [51]. 

In view of that, according to the characterisation of the microstructure of grouts prepared using a sulphate-resisting Portland cement and grouts with silica fume addition in this research, it could be confirmed the hypothesis exposed in the abovementioned previous works [24,26,35]. This hypothesis argued that mercury intrusion porosimetry, which is the most commonly used destructive technique for the pore network characterisation of cementitious materials, would not be useful to follow the evolution of sulphate attack in those materials, while the new non-destructive impedance spectroscopy technique allow the changes in the pore network of the grouts after such an attack to be studied with greater accuracy.

Lastly, considering the study of the microstructure of the analysed grouts, and despite the differences among the techniques used, all of them agreed that grouts for micropiles that incorporate 5% and 10% of silica fume, have similar or even better performance in the very long-term in comparison with grouts prepared using sulphate-resisting Portland cement.

The period of study also showed a continuous rise in the mass variation of the samples, due to contact with sodium sulphate solution, as seen in Figure 10. According to these results, for both silica fume grouts and those prepared with CEM I SR, the sulphate attack would not produce notable damages on the macroscopic scale, at least with respect to the loss of material, in spite of detected effects on the microstructural scale.

Finally, the general growth of the compressive strength for the grouts in the short-term, as seen in Figure 11, could be related to the formation of new solids as products of clinker hydration and silica fume pozzolanic reactions, which has been previously explained and would be in keeping with microstructure characterisation results. The compressive strength reduction noted at later exposure stages for all the studied grouts could be due to the deleterious effects of sulphate attack, which also coincides with impedance spectroscopy results. Nevertheless, at 365 and 600 days, the compressive strength of silica fume grouts was very similar to that observed for CEM I SR grouts. Therefore, the grouts with a content of silica fume up to 10%, hardened in contact with a sodium sulphate aggressive medium, showed good behaviour in relation to compressive strength in the very long-term, in comparison with sulphate-resisting Portland cement grouts.

## 5. Conclusions

The main conclusions that can be drawn from the results previously discussed can be summarised as follows:

- The microstructure of 5% and 10% silica fume cement grouts exposed to sodium sulphate medium was more refined than that noted for sulphate-resisting Portland cement grouts during the entire studied period (up to 600 days). Moreover, the pore network of the grouts became more refined as the percentage of silica fume increased.
- In the short-term, a progressive pore refinement was observed in all the studied grouts, independently of cement type used. This has been related to the development of clinker hydration and silica fume pozzolanic reactions, which form new solid phases.
- The faster increasing rate of the majority of the impedance spectroscopy parameters observed in silica fume grouts, as well as the quicker pore refinement process, could be related to the possible role of Na_2_SO_4_ as an activator of the pozzolanic reactions of this addition, which would accelerate the new solids formation.
- The differences between the impedance spectroscopy resistances R_1_ and R_2_ results in the long-term could be due to the different deleterious effects produced by sulphate attack in the percolating pores, and in the occluded pores of the pore network of the grouts.
- The fall at later ages of the majority of the impedance spectroscopy parameters, as well as compressive strength for all the analysed grouts could be probably due to the sulphate attack development.
- In view of the results obtained, apparently the non-destructive impedance spectroscopy would be the most sensitive technique for detecting the processes developed during the sulphate attack in the microstructure of cement grouts for micropiles with a content of silica fume up to 10% and for grouts prepared with sulphate-resisting Portland cement grouts in the very long-term (up to 600 days). This would confirm the good results obtained in a previous work using this technique for grouts with slag and fly ash exposed to the same aggressive conditions.
- The Wenner four-point electrical resistivity test and mercury intrusion porosimetry seems to have drawbacks when they are used to measure changes in the microstructure of cement grouts following sulphate attack.
- Despite noting the microstructural effects of sulphate attack in the studied grouts, apparently it would not create notable damages at the macroscopic scale in the very long-term (600 days), at least with respect to the loss of material.
- The compressive strength of silica fume grouts was very similar to that observed for sulphate-resisting Portland cement grouts at later ages (365 and 600 days) of exposure to the sulphate aggressive medium.
- According to the results obtained in this research, micropiles grouts with a content of silica fume up to 10% and exposed to an aggressive medium with a high content of sulphates, have a similar or even better behaviour in the very long-term (600 days), in comparison with grouts prepared using sulphate-resisting Portland cement.
